# Structural Plasticity in Human Heterochromatin Protein 1β

**DOI:** 10.1371/journal.pone.0060887

**Published:** 2013-04-09

**Authors:** Francesca Munari, Nasrollah Rezaei-Ghaleh, Shengqi Xiang, Wolfgang Fischle, Markus Zweckstetter

**Affiliations:** 1 Department for NMR-Based Structural Biology, Max Planck Institute for Biophysical Chemistry, Göttingen, Germany; 2 German Center for Neurodegenerative Diseases (DZNE), Göttingen, Germany; 3 Laboratory of Chromatin Biochemistry, Max Planck Institute for Biophysical Chemistry, Göttingen, Germany; University of Leeds, United Kingdom

## Abstract

As essential components of the molecular machine assembling heterochromatin in eukaryotes, HP1 (Heterochromatin Protein 1) proteins are key regulators of genome function. While several high-resolution structures of the two globular regions of HP1, chromo and chromoshadow domains, in their free form or in complex with recognition-motif peptides are available, less is known about the conformational behavior of the full-length protein. Here, we used NMR spectroscopy in combination with small angle X-ray scattering and dynamic light scattering to characterize the dynamic and structural properties of full-length human HP1β (hHP1β) in solution. We show that the hinge region is highly flexible and enables a largely unrestricted spatial search by the two globular domains for their binding partners. In addition, the binding pockets within the chromo and chromoshadow domains experience internal dynamics that can be useful for the versatile recognition of different binding partners. In particular, we provide evidence for the presence of a distinct structural propensity in free hHP1β that prepares a binding-competent interface for the formation of the intermolecular β-sheet with methylated histone H3. The structural plasticity of hHP1β supports its ability to bind and connect a wide variety of binding partners in epigenetic processes.

## Introduction

Proteins of the Heterochromatin Protein 1 (HP1) family are important regulators of chromatin structure and function in almost all eukaryotes. The human genome encodes three HP1 isoforms, hHP1α, hHP1β and hHP1γ with distinct sub-nuclear localization and potential activity [1]. HP1α and HP1β are mainly found at heterochromatin sites where they mediate chromatin condensation and gene silencing. HP1γ has also been found in euchromatin domains where it seems to be involved in the expression of active genes [2]. The pivotal role of HP1 proteins in genome regulation and their putative connection to the development of cancer [3] have motivated increasing efforts to understand the molecular basis of their biological activity.

HP1β is of particular interest as it is the only isoform essential for viability in mammals [4]. This protein has a multi-domain organization common to the whole HP1 family. A long weakly conserved hinge region links two globular modules, the chromodomain (CD) and the chromoshadow domain (CSD) at the N- and C-terminal sides, respectively. Two additional highly charged regions constitute the N- and C-terminal tails. CD is a “histone post-translational modification (PTM) reader”. A binding pocket characterized by a conserved aromatic cage selectively discriminates the methylation states of Lys9 in histone H3 [5]–[7]. The tri-methylated form (H3me3K9) of this chemical modification is one of the most studied epigenetic marks associated with gene silencing [8]. The NMR structure of CD [9] has revealed a globular shape made of a three-stranded anti-parallel β-sheet packed against a C-terminal α-helix. In the complex, the histone H3 peptide acquires an extended conformation and forms an intermolecular β-sandwich with CD [5]. The CSD has a similar fold with the key difference of two α-helices at the C-terminus that constitute an interface for HP1 dimerization [10]. The CSD dimerization interface provides an additional binding platform for diverse protein partners containing a common PXVXL motif [11]. For HP1a, it was shown that the C-terminal tail cooperates with CSD to discriminate the binding among different partners [12]. HP1, via the CSD, can bind proteins from different biological pathways such as transcriptional repression (KAP1) [10], [13], chromatin assembly (CAF1) [14], nucleosome remodeling (ATRX) [15], nuclear lamina organization (LBR) [16] and DNA replication (ORC) [17]. The ability of CD and CSD to recruit protein partners from diverse biological networks makes HP1 a powerful molecular connector of different cellular pathways.

The hinge region contains a nuclear localization sequence and has the most variable amino acid sequence among human HP1 isoforms and HP1 from different species. It has been reported to be highly accessible to proteases [9] and it was suggested to be unstructured [10]. Chemical modifications on the linker, especially phosphorylation [18], influence HP1 localization, interaction and function in *Drosophila melanogaster*. Beyond the basic CD-CSD connection function, the hinge region seems therefore of functional relevance in tuning HP1 activity. Moreover, for some HP1 isoforms, it can bind DNA [19], [20]. Recently we showed that interactions of the hinge region and the N-terminal tail with DNA mediate the weak association of hHP1β to unmodified nucleosomes, thus providing an alternative mechanism of chromatin binding besides the specific recognition of methylated histone H3 by the CD [21]. Similarly, the N-terminal tail is weakly conserved and contains residues available for post-translational modifications that can modulate HP1 binding to chromatin [22].

The bifunctional and dimeric nature of HP1 appears to be the key for its biological function. While structures have been deposited for the isolated CD and CSD, no detailed information is available for the full-length protein. In particular, little information about the conformational propensities and dynamics of the hinge region and the long N- and C-terminal tails is available. We therefore used NMR spectroscopy to investigate the structural and dynamical properties of both the non-globular and folded domains in full-length human HP1β. Our study reveals both inter-domain motions and internal dynamics in CD and CSD that can promote complex formation with different binding partners.

## Results and Discussion

### hHP1β Populates an Extended Ensemble

**Figure 1:**
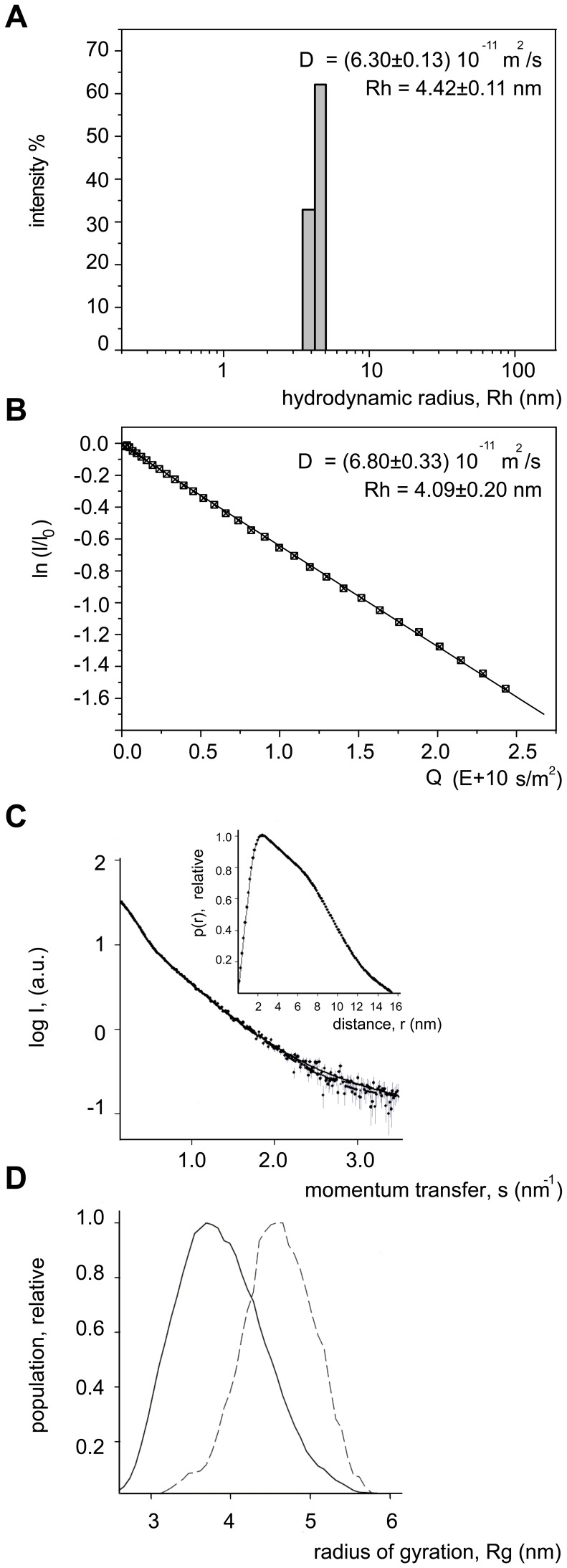
hHP1β populates an extended ensemble. **A.** Dynamic light scattering of hHP1β. The histogram plot shows the experimental data from one measurement consisting of 20 acquisitions. The diffusion coefficient value (D) reported is an average of five measurements done at identical conditions. **B.** PFG-NMR based diffusion plot of hHP1β. The natural logarithm of the intensity ratio I/I_0_ linearly correlates (R = 0.98) with Q, a combined parameter dependent on the gradients strength and delays as defined in [51]. For the intensity ratio, four integrated signals in the 2.4–0.7 ppm region were measured from 32 spectra recorded with increasing gradient strength from 5–75% of the maximum value. Diffusion coefficient values (D) from NMR and DLS experiments were converted into hydrodynamic radius (R_h_) values based on the Stokes-Einstein’s equation. **C.** Small angle X-ray scattering profile of hHP1β. The plot displays the decimal logarithm of the scattering intensity as a function of momentum transfer, s. The distance distribution function is displayed in the inset. **D.** *R_g_* distributions from EOM for hHP1β: initial random pool (continuous line) and selected ensembles averaged over 50 independent EOM runs (dashed line).

To obtain insight into the global conformation of the 184-residue full-length hHP1β in solution we investigated its hydrodynamic behaviour. Dynamic light scattering measurements resulted in a well-defined monodisperse peak with a hydrodynamic radius of 4.4±0.1 nm (figure 1A). The value is in agreement with the results from pulse field gradient NMR (figure 1B) and indicates that the protein does not assume a compact state in solution.

SAXS experiments were then performed to obtain information about the size and shape of the conformational ensemble populated by hHP1β in solution. For hHP1β at 1.0–5.0 mg/mL concentrations, we obtained a MW of 40±4 kDa and an excluded volume of the particle of 85±5 nm^3^. The data confirmed that the protein is present in a dimeric state within the range of tested concentrations. Values of the radius of gyration (R_g_) and maximal size of the particle (D_max_) (4.7±0.2 nm and 15.5±0.5 nm, respectively) as well as the long tail of the distance distribution function p(r) (figure 1C, inset) pointed to a relatively elongated shape of hHP1β. To take into account the dynamic nature of hHP1β, SAXS data were also subjected to the ensemble optimization method, which is particularly suited for flexible multi-domain proteins as it accounts for multiple configurations of disordered linkers [23]. Comparison of the *R*_g_ distribution derived from the optimized ensembles with that obtained from a pool of randomly generated models is shown in figure 1D. The *R*_g_ distribution of the selected ensemble is nearly as broad as the one from the initial random pool, with the maximum shifted towards longer distances, indicating that the hinge region is highly flexible with a preference for more extended conformations.

### CD and CSD do not Form Stable Inter-domain Contacts in Full-length hHP1β

**Figure 2:**
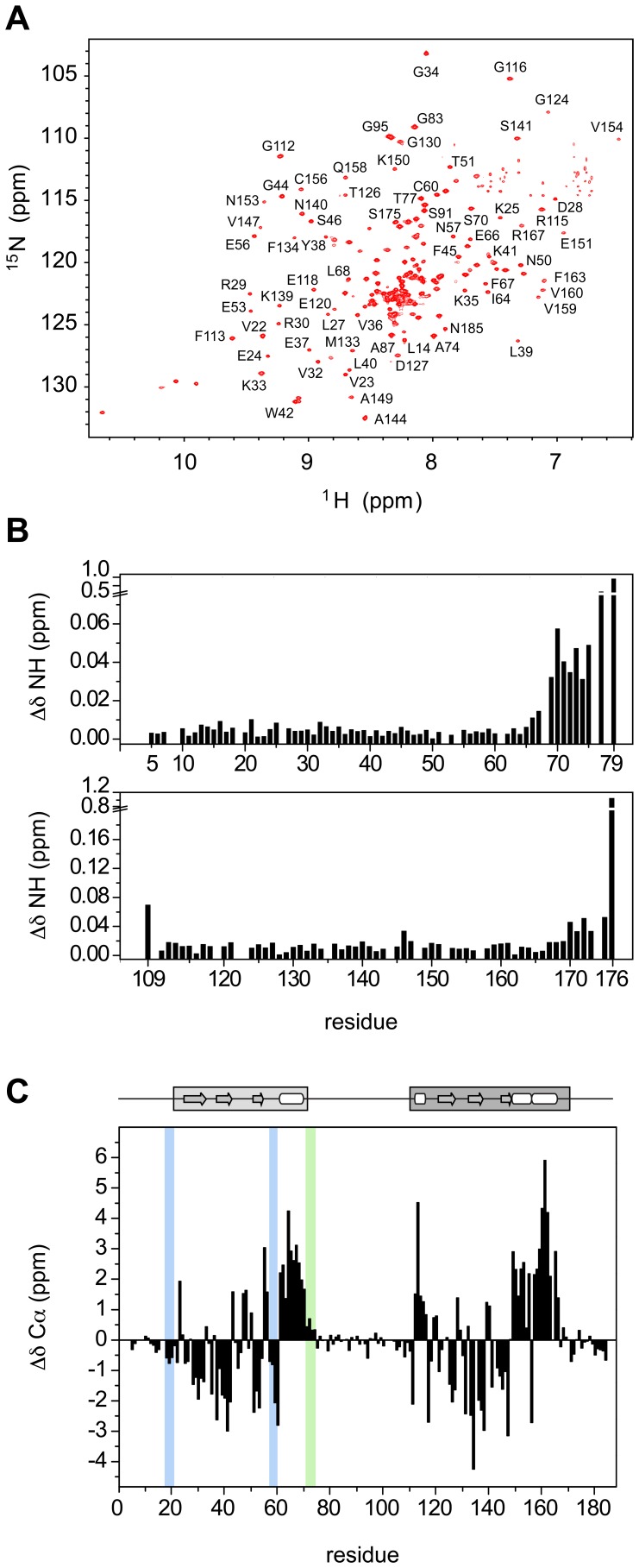
Structural properties of full-length hHP1β. **A.** ^1^H-^15^N TROSY-HSQC spectrum of ^15^N-labelled perdeuterated hHP1β recorded at 303 K on 900 MHz. Selective resonance assignments are indicated. **B.** Average ^1^H-^15^N chemical shift differences (ΔδNH) between CD in full-length hHP1β (2–185) and in the short mutant (2–79) (upper panel). Average ^1^H-^15^N chemical shift differences between CSD in full-length hHP1β (2–185) and in the isolated domain (107–176) (lower panel). **C.** Secondary Cα chemical shifts (ΔδCα) as a function of residue number. The average uncertainty threshold is estimated as 0.1 ppm for the non-globular parts, 0.2 ppm for CD and 0.3 ppm for CSD due to different relaxation properties of these regions. In CD and CSD the presence of segments of continuous positive and negative secondary Cα shifts identifies, respectively, the helix and β-sheet elements in agreement with the secondary structure, schematically shown at the top, as defined in 1AP0 [9] and 1DZ1 [10] PDB files and definition by DSSP [58]. Blue (extended) and green (helical) stripes highlight the additionally identified secondary structure propensities.

The ^1^H-^15^N TROSY-HSQC spectrum of ^15^N-perdeuterated hHP1β (figure 2A) highlights its multi-domain nature. Besides the two structurally related CD (21–71) and CSD (110–170), the remainder of the protein, which accounts for more than one third of the sequence, has a non-globular character with a high percentage of charged residues. The non-globular nature of the hinge region and the N- and C-terminal tails lead to severe signal overlap between 8 and 8.5 ppm in the ^1^H dimension and a large dynamic range of peak intensities. In particular, the clustering of lysine and glutamate residues in the sequence complicates the sequence-specific resonance assignment by conventional triple-resonance techniques, thus precluding the structural characterization of the full-length protein so far. By using automated projection spectroscopy (APSY) experiments on hHP1β (2–185), in combination with the use of protein fragments, we were able to assign most of the hHP1β backbone signals [21].

Analysis of the average ^1^H-^15^N chemical shift differences between CD in hHP1β (2–185) and the isolated CD (residues 2–79) (figure 2B, upper panel), and between CSD in hHP1β (2–185) and the isolated CSD (residues 107–176) (figure 2B, lower panel), suggested that the structures of both domains are retained in the full-length protein. Moreover, the lack of chemical shift changes except at the termini of the domains excludes significant inter-domain contacts between CD and CSD, in line with the conclusions reported by Brasher et al. [10]. The mutual independence of the two domains was further supported by ^1^H-^15^N residual dipolar coupling (RDC) analysis. Alignment tensors calculated from experimental RDCs and 3D structures had different magnitudes: 9.67 Hz for CD and 29.54 Hz for CSD.

### Structural Propensities in the Non-globular Domains

The conformational properties of the hinge region and of the N- and C-terminal tails were addressed by analysis of NMR secondary chemical shifts, that are highly sensitive probes of local conformation [24]. For most residues in the tails and the hinge region the absolute values of Cα secondary chemical shifts were below 0.3 ppm (figure 2C and, for the combined secondary chemical shifts see figure S1), supporting their intrinsically disordered nature. In the hinge region a weak helical tendency expands the α-helix of the CD beyond residue 70 up to 73. In addition, a continuous stretch of small negative Cα secondary shifts in proximity to residue 20 points to a propensity for extended conformations in the N-terminal tail. The extended conformation in this region might be favored by the high density of charged residues and may be functionally relevant for formation of the β-sheet sandwich between strand β1 of the CD and the induced β-strand in methylated histone H3. In line with this hypothesis, β1 is N-terminally extended in the dmHP1CD-methylated histone H3 complex [5].

### Modular Dynamics of hHP1β

**Figure 3:**
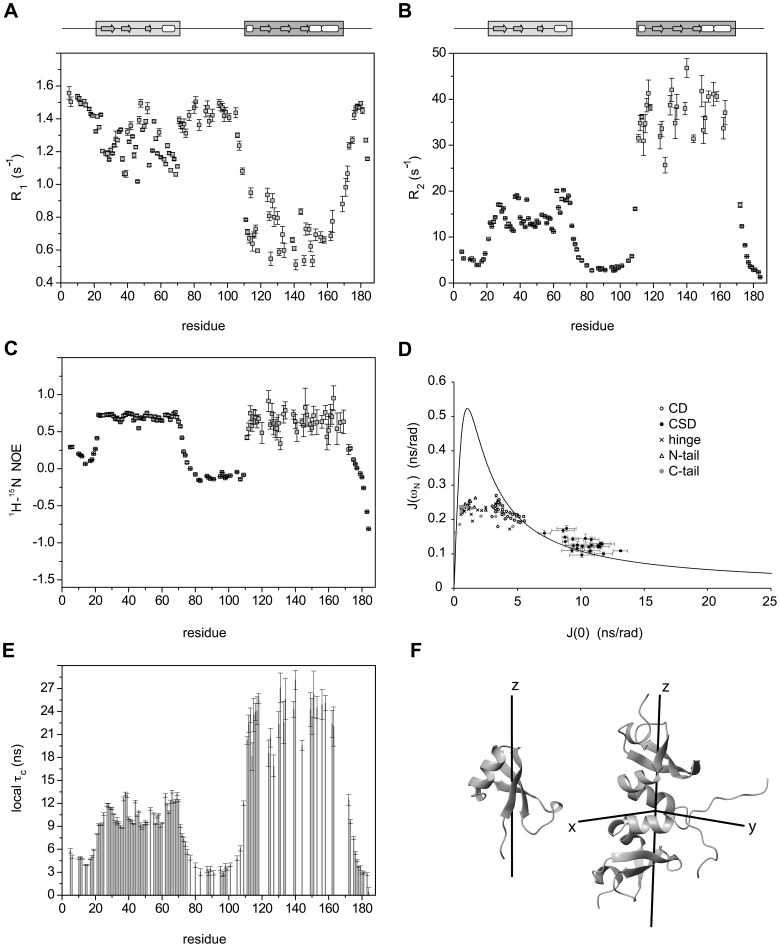
Backbone dynamics probed by ^15^N- relaxation rates. **A, B, C.** ^15^N spin-relaxation rates for ^15^N-perdeuterated hHP1β measured at a proton Larmor frequency of 600 MHz at 298 K. Residues with severe peak overlap or insufficient signal-to-noise ratio were excluded. R_1_ (A), R_2_ (B) and steady-state ^1^H-^15^N heteronuclear NOE (C) are shown along the protein sequence. The R_2_ rates were derived from R_1ρ_ measurements upon correction for the off-resonance tilted field as described in the Methods. **D.** Graphical analysis of reduced spectral density mapping. The solid line represents the theoretical function of J(ω_N_) versus J(0) assuming a rigid single Lorentzian motion. Experimental values are plotted as points with labels specific for each hHP1β domain. **E.** The rotational correlation time τ_c_, determined for each residue from the R_2_/R_1_ ratio, is shown as a function of residue number. **F.** Illustration of the diffusion tensor of CD (left, axially symmetric) and CSD (right, fully anisotropic) in full-length hHP1β. The x- and y-axes are shown as half-axes only for the fully anisotropic tensor. Pictures were prepared using MolMol [57].

To obtain insight into the dynamic properties of hHP1β, we performed ^15^N spin relaxation measurements [25]. In the globular CD and CSD the average R_1_ (R_2_) relaxation rate was 1.24±0.11 s^−1^ (14.85±2.62 s^−1^) and 0.71±0.11 s^−1^ (37.01±4.54 s^−1^), respectively (figures 3A–B). In addition, average hetNOE values of 0.70±0.04 for CD and 0.64±0.13 for CSD (figure 3C) indicated that the protein backbone is very rigid in the two domains. From the R_2_/R_1_ ratios average τ_c_ values of 10.68±1.45 ns and 23.47±2.41 ns were calculated for the CD and CSD, respectively. Residue-specific τ_c_ values in the C-terminal α-helical region of CD were consistently higher than the average τ_c_ of the domain (figure 3E), indicating that the global motion of CD is anisotropic. Also CSD shows features of anisotropic global motion: the product of R_1_ and R_2_ (R_1_R_2_) efficiently removes the anisotropy that causes a large distribution of R_2_/R_1_ [26] (figure S2A).

To characterize the anisotropy of the global motion of CD and CSD within full-length hHP1β, we determined the rotational diffusion tensor of the two domains using the program ROTDIF [27] (Table 1). The best fit for CD was obtained with the axially-symmetric diffusion model (Q = 0.35), resulting in average values for τ_c_ and anisotropy of 10.19±1.18 ns and 2.02±0.38, respectively. The orientation of the diffusion tensor of CD is illustrated in figure 3F. The C-terminal helix of CD is aligned nearly parallel to the z-axis of the diffusion tensor, providing a rationale for its large τ_c_ values. The CSD data were best fit using the fully-anisotropic diffusion model (Q = 0.37) (Table 1 and figure 3F) with average τ_c_ of 24.57±4.95 ns. The anisotropy and rhombicity of the diffusion tensor were 3.55±1.41 and 0.37±0.13, respectively.

**Table 1:** Rotational diffusion tensor of CD and CSD within full-length hHP1β.

	*Chromodomain*			*Chromoshadow domain*		
model	isotropic	axially symmetric	fully anisotropic	isotropic	axially symmetric	fully anisotropic
**τ_c_ (ns)**	11.11±0.31	10.19±1.18	–	23.36±0.62	23.86±4.55	24.57±4.95
**D_xx_(10^7 ^s** ^−**1**^ **)**	1.50±0.04	1.22±0.08	–	0.71±0.02	0.37±0.01	0.25±0.13
**D_yy_(10^7 ^s** ^−**1**^ **)**	1.50±0.04	1.22±0.08	–	0.71±0.02	0.37±0.01	0.48±0.17
**D_zz_(10^7 ^s** ^−**1**^ **)**	1.50±0.04	2.46±0.50	–	0.71±0.02	1.35±0.40	1.30±0.35
**Q**	0.71	0.35	0.32	0.72	0.60	0.37
**P**		<0.000001	0.32		<0.001	<0.001

Diffusion tensor parameters for the different tumbling models, obtained from experimental ^15^N spin-relaxation data through ROTDIF, are listed: the overall rotational correlation time τ_c_; D_xx_, D_yy_ and D_zz_ are the principal values of the diffusion tensor; Q is the quality factor defined as in [27]; P defines the probability that an improvement in the fit when a more complex model is applied has occurred by chance. The best model for CD and CSD is marked in bold. The little improvement in the fit with the more complex fully-anisotropic model was not statistically significant for CD.

Next we compared the rotational correlation times for the two globular domains in full-length hHP1β to values predicted for the isolated domains using the program HYDROPRO [28]. HYDROPRO estimated the τ_c_ values of the isolated CD and the isolated dimeric CSD as 4 ns and 10 ns, respectively. Thus, rotational correlation times in the isolated domains are more than a factor of two smaller than in full-length hHP1β. The strong increase in τ_c_ for the two globular domains in the full protein points to the presence of motional coupling. With the apparent lack of any persistent structure in the intervening hinge region or any stable contact between the two domains, this motional coupling seems to be mainly contributed by hydrodynamic interaction: in the spatial proximity of the other domains, tumbling of each domain is slowed down in comparison with the isolated state as a consequence of a stronger resistance to the accompanied solvent displacement. The presence of hydrodynamic coupling has been demonstrated for several multi-domain proteins, for example a two-domain model protein [29], and appears to be a generic feature of modular proteins with flexible linkers.

According to ^15^N spin relaxation rates the backbone outside of CD and CSD is highly mobile (figure 3). The high mobility of these regions precludes the analysis of their dynamic properties through separation of global and internal motions. Therefore, we analyzed ^15^N spin relaxation rates by reduced spectral density mapping to describe protein NH vector motions at time scales corresponding to three different frequencies 0, ω_N_ and 0.87ω_H_ [30] (figure 3D and figure S3). The N- and C-terminal tails as well as the hinge region showed much smaller J(0) and bigger J(0.87ω_H_) than the two globular domains, indicating a very slow decay of their spectral density function characteristic of fast tumbling molecules with high internal mobility. The J(0.87ω_H_) profile demonstrated that the N-terminal tail experiences smaller internal dynamics when compared to the hinge region, with the C-terminal tail being the most flexible part among the disordered domains (figure S3A). A reduced mobility on the pico-to-nanosecond time scale in the N-terminal tail is further supported by an average hetNOE value of 0.18±0.08. Interestingly, the N-terminal tail is more rigid at its beginning up to residue L14, where the hetNOE reaches a local minimal value of 0.06, and then starts to rise afterwards (figure 3C).

### Internal Dynamics in the Binding Pockets of CD and CSD

**Figure 4:**
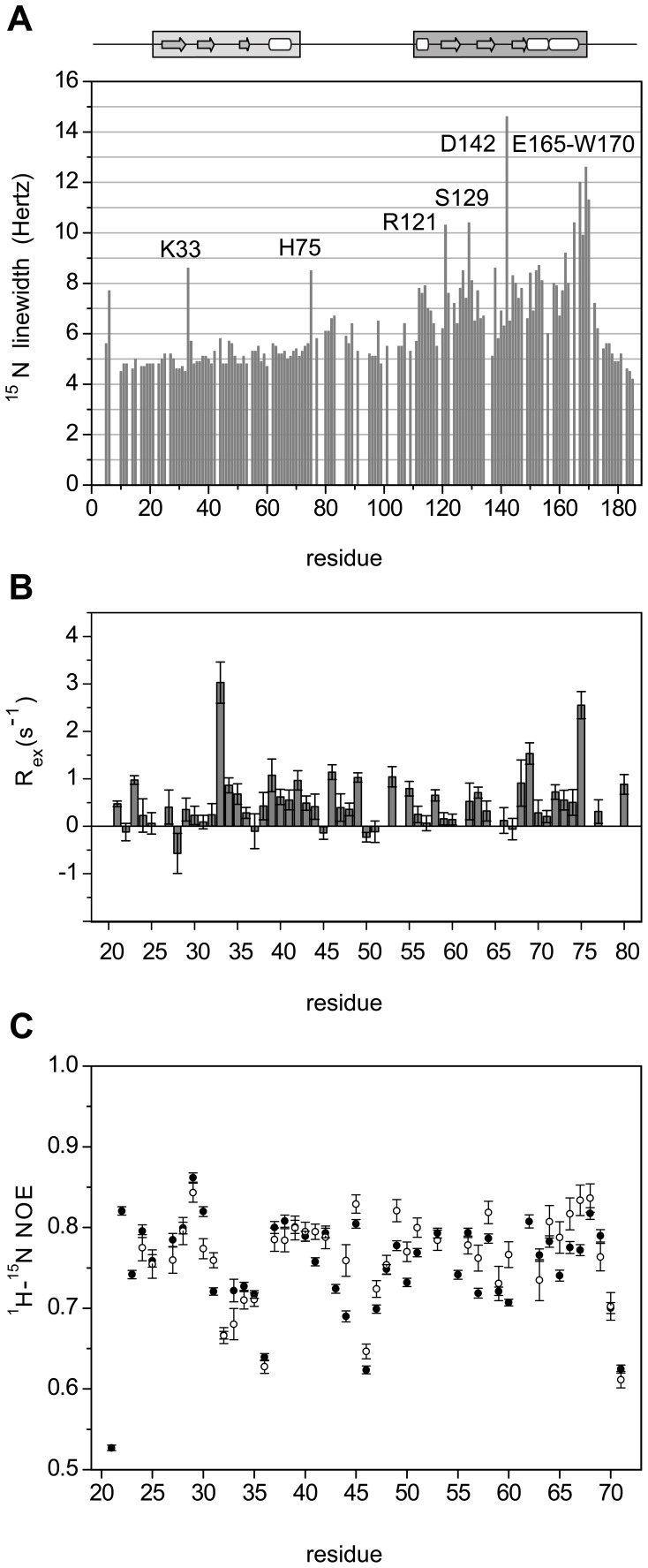
Internal dynamics. **A.** ^15^N linewidths of perdeuterated hHP1β as a function of residue number, measured from a TROSY-HSQC recorded at 700 MHz and 303 K. **B.** R_ex_ values of CD in full-length hHP1β as a function of residue number. **C.** Comparison of steady-state ^1^H-^15^N heteronuclear NOE values of CD in full-length hHP1β in the free state (black circles) with those of CD in full-length hHP1β in complex with the H3K_C_9me3 peptide (1–15) at a molar ratio of 1∶4 (white circles). Both measurements were performed at 298 K, 600 MHz proton Larmor frequency, 5 s recycle delay, on a 0.3 mM ^15^N-perdeuterated hHP1β sample.

Internal dynamics play a key role for molecular recognition [31]. Therefore, we investigated the internal motions in CSD and CD, the two domains that mediate binding of HP1 to a wide variety of protein partners. In CSD, residues E165 to W170 showed the largest ^15^N linewidths (figure 4A). Besides a possible local effect due to H171, the observed signal broadening points to slow conformational rearrangements. Importantly, this region is involved in interactions with different proteins, suggesting that this conformational plasticity may support interaction with PXVXL motif and its variants in CSD binding partners.

In CD, which is essential for binding to methylated histone H3 in chromatin, strong signal broadening (figure 4A) and slow chemical exchange (slower than ∼100 µs) (figure 4B) was observed for H75, close to the hinge region, and K33, which is located in the short loop between β-strands β1 and β2. The mobility of the loop comprising K33 was further supported by a prominent exchange contribution to the ^15^N R_2_ relaxation rate and distinct temperature sensitivity of its peak intensity (figure S4). Besides K33, peak intensities of four residue stretches in CD (L27–D28; E56–C60; L63–I64; Q69–S70) showed pronounced temperature sensitivity that points to a change in the flexibility of these regions as a function of temperature (figure S4C).

### N57 to C60 of the CD Populate Binding-competent Conformations Prior to Binding to Methylated Histone H3

**Figure 5:**
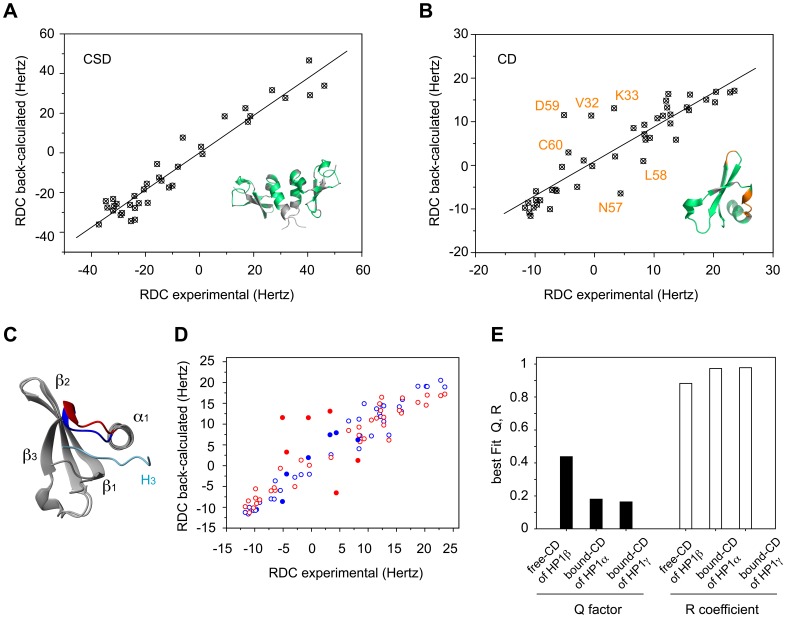
Analysis of CD and CSD structures by RDCs. **A.** Correlation between experimental ^1^H-^15^N RDCs and values back-calculated from the 3D structure of CSD of hHP1β (PDB code: chain A, 2FMM [59]) using singular value decomposition method. The analysis comprised 36 residues, spanning R111–F163, that are marked in green on the model structure. **B.** Correlation between experimental ^1^H-^15^N RDCs and values predicted from the 3D structure of CD of hHP1β (PDB code: 3F2U). The 45 residues analyzed, spanning Y21–Q69, are marked in green on the model structure. Outliers are marked in orange. In A and B, residues affected by overlap or with insufficient signal-to-noise ratio were excluded from the analysis. **C.** Alignment between free CD of hHP1β (PDB code: 3F2U) and bound CD of hHP1α (PDB code: 3FDT). The β3-α1 intervening region that includes the N57–C60 residues (outliers in the RDCs analysis) is shown in red for free CD and in blue for bound CD. The histone peptide of 3FDT PDB is coloured in light blue. **D.** Correlations between experimental ^1^H-^15^N RDCs and values predicted from the 3D structure of free CD of hHP1β (PDB code: 3F2U) (red) or from bound CD of hHP1α (PDB code: 3FDT) (blue). Residues showing a remarkably different correlation among the compared structures are highlighted as filled circles. **E.** Comparison of the fitting quality parameters, Q and R, from analysis of experimental RDCs using different X-ray structures of CD.

How is the structure of CD and CSD in solution affected by the observed conformational dynamics? To address this question, we measured residual dipolar couplings that describe the orientation of internuclear vectors and are therefore highly sensitive probes of structure and dynamics [32]. Experimental ^1^H-^15^N RDCs of CSD in full-length hHP1β correlated well with values back-calculated from the X-ray structure of CSD (Pearson’s correlation coefficient R = 0.97 and dipolar coupling quality factor Q = 0.15) (figure 5A). The fit of experimental RDCs to the X-ray structure of the CD was of much lower quality with R = 0.89 and Q = 0.44. In particular, six RDC values deviated significantly. Their removal improved the quality of the fit to R = 0.98 and Q = 0.18 (figure 5B). The six RDC values belong to the two residue stretches V32–K33 and N57–C60, which experience a wide range of internal motions (figure 4 and figure S4).

Inspection of the 3D structure of CD reveals that N57–C60 is part of the intervening region between strand β3 and helix α1 (figures 5B and C). In the X-ray structure of the isolated, unbound CD, a helical turn following the β3-strand has been assigned to residues E56–L58 (PDB code: 3F2U [33]). However, NMR secondary chemical shifts (marked in figure 2C and figure S1A–C) rather point to a propensity for extended conformation in the L58–D59 region. Thus, crystallization may have stabilized the β3-α1 intervening region in a conformation that is weakly represented in solution providing a rationale for why N57–C60 coordinates from the X-ray structure are not compatible with experimental RDCs. Upon interaction with methylated histone H3, residues L58–D59 are stabilized in a well-ordered intermolecular β-sandwich, as shown for CD of hHP1α (PDB code: 3FDT [6], displayed in figure 5C in comparison with 3F2U structure), hHP1β (PDB code: 1GUW [7]) and dmHP1 (PDB code: 1KNE [5]). We therefore tested whether our experimental ^1^H-^15^N RDCs better fit to those back-calculated from the available crystal structures of bound CD (figure 5D–E and figure S1D). A best fit of 43 experimental RDCs spanning V23–Q69 resulted in R = 0.88 and Q = 0.44 when using the PDB of free-CD of hHP1β (PDB code: 3F2U), R = 0.97 and Q = 0.18 with the PDB of bound-CD of hHP1α (PDB code: 3FDT), and R = 0.98 and Q = 0.16 with the PDB of bound-CD of hHP1γ (PDB code: 3TZD [34]). The strong improvement in the quality of the RDC fit supports the presence of a binding-competent conformation for N57–C60 in the CD prior to interaction with methylated histone H3, where L58–D59 have already a propensity for more extended conformation. Upon binding, N57–C60 become more rigid as evidenced by an increase of hetNOE values in this region in the complex of hHP1β with the H3K_C_9me3 peptide (figure 4C).

### Conclusions

Here we showed that hHP1β explores a wide conformational space by populating an extended ensemble. Due to the high flexibility of the hinge region the two globular domains, CD and CSD, remain independent. The absence of direct contacts enables a largely unrestricted spatial search by the two domains for their binding partners. This feature is of primary importance in the activity of HP1 to bridge nearby nucleosomes to form heterochromatin [21], [35], [36], and to recruit and connect different binding partners belonging to diverse pathways related to chromatin function [37]. In addition, the intrinsic disorder of the hinge region facilitates post-translational modifications by enzymes such as protein kinases [18], [38], makes the linker an accessible hold for interaction with nucleic acids [18], [20], [21], [39] and increases the exposure of the nuclear localization sequence for proper localization [37]. Our data moreover reveal that, despite the high flexibility of the hinge region, the rotational diffusion of both CD and CSD is evidently slowed down by the presence of other domains within the full-length protein. Thus, hHP1β provides an example of how hydrodynamic interaction alters the tumbling of domains within flexible modular proteins.

Both CD and CSD have distinct structural and motional properties in regions important for molecular recognition. In CSD, strong signal broadening between E165 and W170 points to the presence of slow conformational rearrangements that can be useful for the versatile recognition of the PXVXL motif and its variants [40]. Moreover, the N57–C60 region, which is stabilized in an intermolecular β-sheet upon interaction of the CD with methylated histone H3, has a propensity for the binding-competent conformation already in free hHP1β. The propensity for extended structure is enforced upon interaction with histone peptide to complete the β-barrel architecture of the domain [41]. Interestingly, structures of the same CD bound to different peptides, such as CD of HP1γ with a histone1.4 peptide and CD of HP1γ with a histone-lysine N-methyltransferase peptide [34], show different backbone geometries in this region, indicating that a certain degree of conformational plasticity could better accommodate different binding partners. In summary, the structural plasticity of hHP1β promotes its activity in binding and connecting a variety of proteins related to epigenetic events.

## Methods

### Production of Recombinant hHP1β Proteins

The following hHP1β sequences (GenBank NM_001127228; UniProt accession number P83916) were cloned into a pET16b expression vector (Novagen) modified with an N-terminal TEV-protease-cleavable His-tag site: full-length (2–185), CD (2–79), CD (19–79) and CSD (107–176). Details of plasmid constructs are available upon request.

hHP1β (2–185) protein for SAXS and DLS experiments and isotope-labeled hHP1β (2–185), CD (2–79), CD (19–79) and CSD (107–176) proteins for NMR measurements were expressed and purified as described previously [21]. An additional size-exclusion chromatography on a Superdex200 column (GE Healthcare) was performed for SAXS and DLS samples.

All samples were finally prepared in 20 mM sodium phosphate (pH 6.5), 50 mM NaCl, 2 mM DTT and 0.02% NaN_3_ and filtered before the experiments.

The H3K_C_9me3 1–15 peptide was obtained from a synthetic peptide H3(1–15)K9C where a Cys residue replaced the Lys at position 9. This modification allowed the site-specific installation of a tri-methyl lysine analog by Cys-alkylation reaction using (2-bromoethyl)-trimethylammonium bromide (Sigma). Details of the alkylation reaction were described previously [21], [42].

### Dynamic Light Scattering

Dynamic light scattering measurements were performed on a Wyatt DynaPro Titan instrument (Wyatt Technology, California) at 303 K on a sample containing 0.1 mM hHP1β.

### SAXS Experiments

Synchrotron radiation X-ray scattering data were acquired on the EMBL X33 beamline at the DORIS III storage ring, DESY, in Hamburg [43]. Experiments were carried out at 283 K, with protein concentrations of 1.0, 2.0 and 5.0 mg/mL. A pixel detector PILATUS 1 M (DECTRIS, Switzerland) at sample-detector distance 2.7 m and wavelength λ = 0.15 nm, covering the momentum transfer range 0.12<s<4.9 nm^−1^ (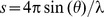 where 2θ is the scattering angle), was employed. Data were processed with the ATSAS program package [44]. For each measurement, four 30 sec exposures were compared to check for radiation damage. No radiation effects were observed. The data were averaged after normalization to the intensity of the incident beam. The signal of the buffer was subtracted and the difference data were extrapolated to zero solute concentration by standard procedures.

Data analysis was performed with the program PRIMUS [45]. The forward scattering I(0) and the radius of gyration R_g_ were obtained using the Guinier approximation, assuming that at very small angles (s<1.3/R_g_) the intensity is represented as 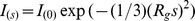. These parameters were also computed from the entire scattering patterns with the program GNOM [46], giving the distance distribution function p(r) and the maximum particle dimension D_max_. The MW was obtained comparing the forward scattering to that from reference solutions of bovine serum albumin (66 kDa). Possible flexibility of hHP1β was assessed by the ensemble optimization method (EOM) [23], which allows for coexistence of different conformations contributing to the experimental scattering pattern. These conformers were selected by a genetic algorithm from a pool containing 10^5^ randomly generated models. Genetic algorithms were employed to find the subsets of the conformers that fit the experimental data best. The obtained subsets were analysed to yield the R_g_ distributions in the optimal ensembles.

### NMR Experiments

NMR spectra for sequence-specific backbone resonance assignment of full-length hHP1β were acquired at 303 K on 600, 700 and 800 MHz Bruker spectrometers equipped with triple resonance cryogenic probes. A combination of standard TROSY-based 3D [47] and APSY 5D, 6D and 7D experiments [48] was used. ^1^H-^15^N assignments were transferred to the CD (2–79), CD (19–79) and CSD (107–176) and verified by additional standard TROSY-based 3D experiments when required.

Secondary chemical shifts were calculated based on the random coil chemical shifts predicted by the Neighbor Corrected Structural Propensity Calculator [49] and corrected for the ^2^H isotope shift. The 4,4-dimethyl-4-silapentane-1-sulfonic acid (0.0 ppm) was used for chemical shift referencing. Consensus chemical shift index (CSI) values were obtained using the RCI webserver [50].

Pulse field gradient stimulated-echo diffusion experiments were performed at 303 K on a sample containing 0.12 mM of hHP1β. Q was defined according to [51]. Diffusion gradient length (little delta) and diffusion delay (big delta) were set to 3 ms and 200 ms, respectively. Gradient calibration was achieved by measuring the diffusion of residual HDO in 99.8% D_2_O at 298 K.

^15^N relaxation experiments were performed at 600 MHz, 298 K, using 0.7 mM ^15^N-perdeuterated hHP1β sample. ^15^N longitudinal relaxation rates (*R*_1_) were measured using relaxation delays of 8, 30, 60, 100, 180, 320, 500, 800 and 1200 ms. ^15^N transverse relaxation rates (“total *R*_2_”) were measured using relaxation delays of 8, 16, 24, 34, 52, 86, 120, 180 and 240 ms. ^15^N longitudinal relaxation rates in the rotating frame (*R*_1*ρ*_) were measured in a near-resonance mode with relaxation delays of 20, 40, 60, 80, 100, 120, 140 and 180 ms and a spin-lock field strength of 2.5 kHz. *R*_1_, *R*_2_ and *R*_1*ρ*_ relaxation rates were determined from the best single exponential fit to the experimental intensity data. Effective R_2_ rates were derived from the relation: 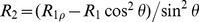, where 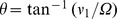, ν_1_ is ^15^N spin-lock field strength (in Hz) and Ω is the resonance offset from the spin-lock carrier (Hz). R_ex_ values were determined as the difference between the “total R_2_” rates and the effective R_2_ rates derived from *R*_1*ρ*_ values. Steady-state ^1^H-^15^N heteronuclear nuclear Overhauser enhancement (NOE) was measured with a total recycle delay of 10 s. NOE values were calculated by the ratio of the peak intensities between saturated and reference spectra. With the reduced spectral density mapping, the relaxation rates R_1_ and effective R_2_ and hetNOEs were transformed to spectral densities at zero frequency J(0), at ^15^N frequency J(ω_N_) and at the effective ^1^H frequency J(0.87ω_H_) [30]. The theoretical relation between J(ω) and J(0) was obtained with the assumption of a single Lorentzian motion, as 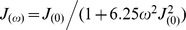.

For rotational diffusion analysis the program ROTDIF7 [27] and the crystal structures of the isolated CD (PDB code: 3F2U) and CSD (PDB code: chain A, 2FMM) were used. For each of the two domains, only residues within the main secondary structural elements were included in the analysis. Three diffusion models, isotropic, axially-symmetric and fully-anisotropic were utilized for calculation of rotational diffusion tensors, and the F-test was applied to evaluate if the improvement of the fit by the more complex model was statistically significant. 500 Monte-Carlo calculations were performed for uncertainty analysis. The R_2_ used in this analysis was the exchange-free R_2_^0^ derived from the ^15^N-^1^H dipole-dipole/^15^N chemical shift anisotropy cross-correlated relaxation rates (η_xy_) according to 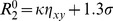, where 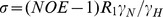. The results with the R_1ρ_-based effective R_2_ were consistent with these results, but had larger uncertainty range for the CSD domain. 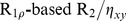, i.e. κ (kappa), was highly similar for the two domains: 1.39±0.05 for CD and 1.41±0.14 for CSD. These values are consistent with a ^15^N CSA magnitude of −162 ppm and an angle of 19.6° between ^15^N CSA and internuclear N–H vector, in line with the recent reports [52]. η_xy_ rates (figure S2B) were measured using relaxation delays (2Δ) of 6, 8, 10, 20 and 60 ms, and determined by signal intensity analysis according to: 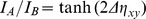, where I_A_ and I_B_ are the signal intensities obtained with pulse schemes A and B in [53], which separately address relaxation of ^15^N downfield and upfield doublet components.

R_1ρ_-based relaxation dispersion experiments for K33 were performed on a sample containing 0.48 mM of ^15^N-labelled CD(19–79) at 303 K and a proton Larmor frequency of 600 MHz. R_1ρ_ rates were obtained at ^15^N spin-lock field strengths of 200, 600, 1000 and 1500 Hz centered on the ^15^N resonance of K33, using six relaxation time delays between 5 and 190 ms.

One bond^ 1^H-^15^N coupling constants of protein backbone were obtained using a TROSY-HSQC interleaved experiment recorded on a 900 MHz NMR spectrometer equipped with a cryoprobe. The sample contained 0.2 mM of ^15^N-perdeuterated hHP1β. The temperature was 293 K. Partial alignment was achieved using a dilute liquid crystalline phase of 5% C12E5/hexanol (Sigma) [54] resulting in 16 Hz of quadrupolar splitting. RDC data were analyzed using the PALES software [55]. Alignment tensors calculated from experimental RDCs and 3D structures (PDB codes: 3F2U and 2FMM, chain A) had magnitudes of 9.67 Hz for CD and 29.54 Hz for CSD with rhombicities of 0.09 and 0.12, respectively.

NMR data were processed with NMRPipe [56] and analyzed with Sparky (T. D. Goddard and D. G. Kneller, University of California, San Francisco). Origin® and Wolfram Mathematica® were used for mathematical and graphical analyses. CD and CSD PDBs were visualized with MolMol [57] and PyMOL (The PyMOL Molecular Graphics System, Version 1.1r1, LLC).

## Supporting Information

Figure S1 **Secondary chemical shifts and RDCs analysis. A, B.** Combined Cα+CO (A) and Cα-Cβ (B) secondary chemical shifts as a function of residue number. The average uncertainty threshold is estimated 0.1 ppm for the non-globular parts, 0.2 ppm for CD and 0.3 ppm for CSD. Blue (extended) and green (helical) stripes highlight the additionally identified secondary structure propensities. The Cα-Cβ secondary chemical shifts have the advantage that they are not affected by any possible imperfection in ^13^C chemical shift referencing. **C.** Consensus chemical shift index (CSI) values for CD from RCI analysis. **D.** Correlations between experimental ^1^H-^15^N RDCs and values predicted from the atomic coordinates of free CD of hHP1β (PDB code: 3F2U) (red) or from bound CD of hHP1γ (PDB code: 3TZD) (green). Residues showing a remarkably different correlation among the compared structures are highlighted as filled circles.
(TIF)

Figure S2 **Analysis of ^15^N spin-relaxation data. A.** The anisotropy of global motion can be detected in the plot of R_2_R_1_ vs R_2_/R_1_ if τ_c_ >>1/ω_N_. The condition is met for CSD where the large distribution of R_2_/R_1_ values along constant R_2_R_1_ denotes an anisotropic rotational diffusion. **B.** Transverse cross-correlated relaxation rates (η_xy_) of hHP1β as a function of residue number.
(TIF)

Figure S3 **Reduced spectral density mapping. A, B, C.** hHP1β ^15^N spin-relaxation rates were analysed by reduced spectral density mapping. Spectral densities at the effective proton frequency J(0.87ω_H_) (A), at the ^15^N frequency J(ω_N_) (B) and at zero frequency J(0) (C) are shown as a function of residue number.
(TIF)

Figure S4 **The slow motion of K33. A.** Relaxation dispersion profile of K33 in ^15^N-labelled CD (19–79). Residue L40 serves as control. **B.** Selected regions of the ^1^H-^15^N TROSY-HSQC spectrum of hHP1β at 283 K (blue), 290 K (light blue), 298 K (green), 303 K (yellow), and 310 K (red). Spectra at different temperatures are displayed with equal counter level. K33 shows strong signal broadening at increasing temperature. **C.** Change of ^1^H-^15^N signal intensity with increasing temperature. The signal intensities at 290 K (blue), 298 K (green), 303 K (yellow), and 310 K (red), relative to the intensity at 283 K, are shown versus the CD sequence.
(TIF)
