# Supplementary figures and images for: Structural Plasticity in Human Heterochromatin Protein 1β
Source: PLoS One. 2013 Apr 9;8(4):e60887. doi: 10.1371/journal.pone.0060887 (PMC3621757; doi:10.1371/journal.pone.0060887)

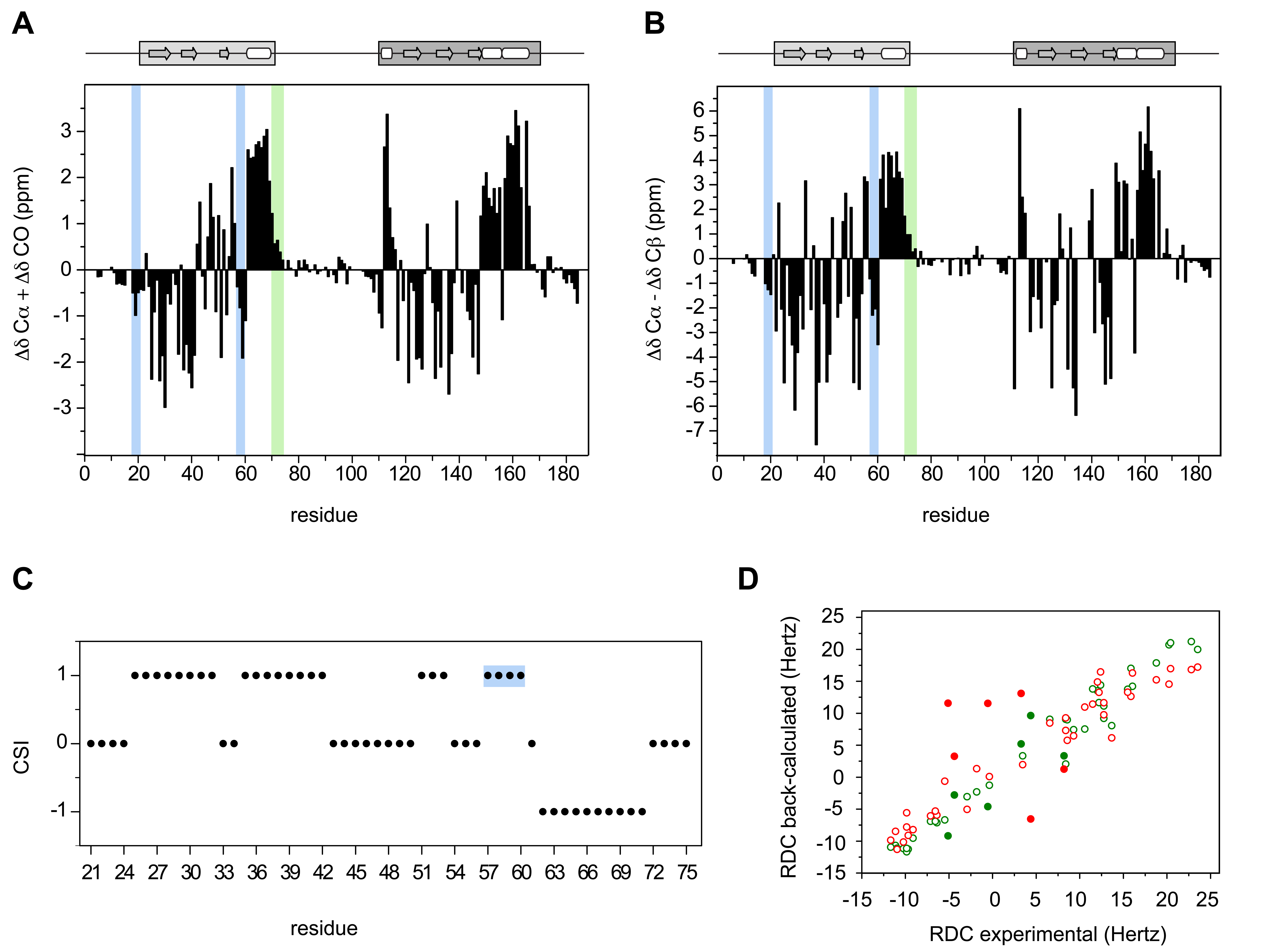

Supplement: Figure S1 — Secondary chemical shifts and RDCs analysis. A, B. Combined Cα+CO (A) and Cα-Cβ (B) secondary chemical shifts as a function of residue number. The average uncertainty threshold is estimated 0.1 ppm for the non-globular parts, 0.2 ppm for CD and 0.3 ppm for CSD. Blue (extended) and green (helical) stripes highlight the additionally identified secondary structure propensities. The Cα-Cβ secondary chemical shifts have the advantage that they are not affected by any possible imperfection in 13C chemical shift referencing. C. Consensus chemical shift index (CSI) values for CD from RCI analysis. D. Correlations between experimental 1H-15N RDCs and values predicted from the atomic coordinates of free CD of hHP1β (PDB code: 3F2U) (red) or from bound CD of hHP1γ (PDB code: 3TZD) (green). Residues showing a remarkably different correlation among the compared structures are highlighted as filled circles. (TIF) [file pone.0060887.s001.tif]

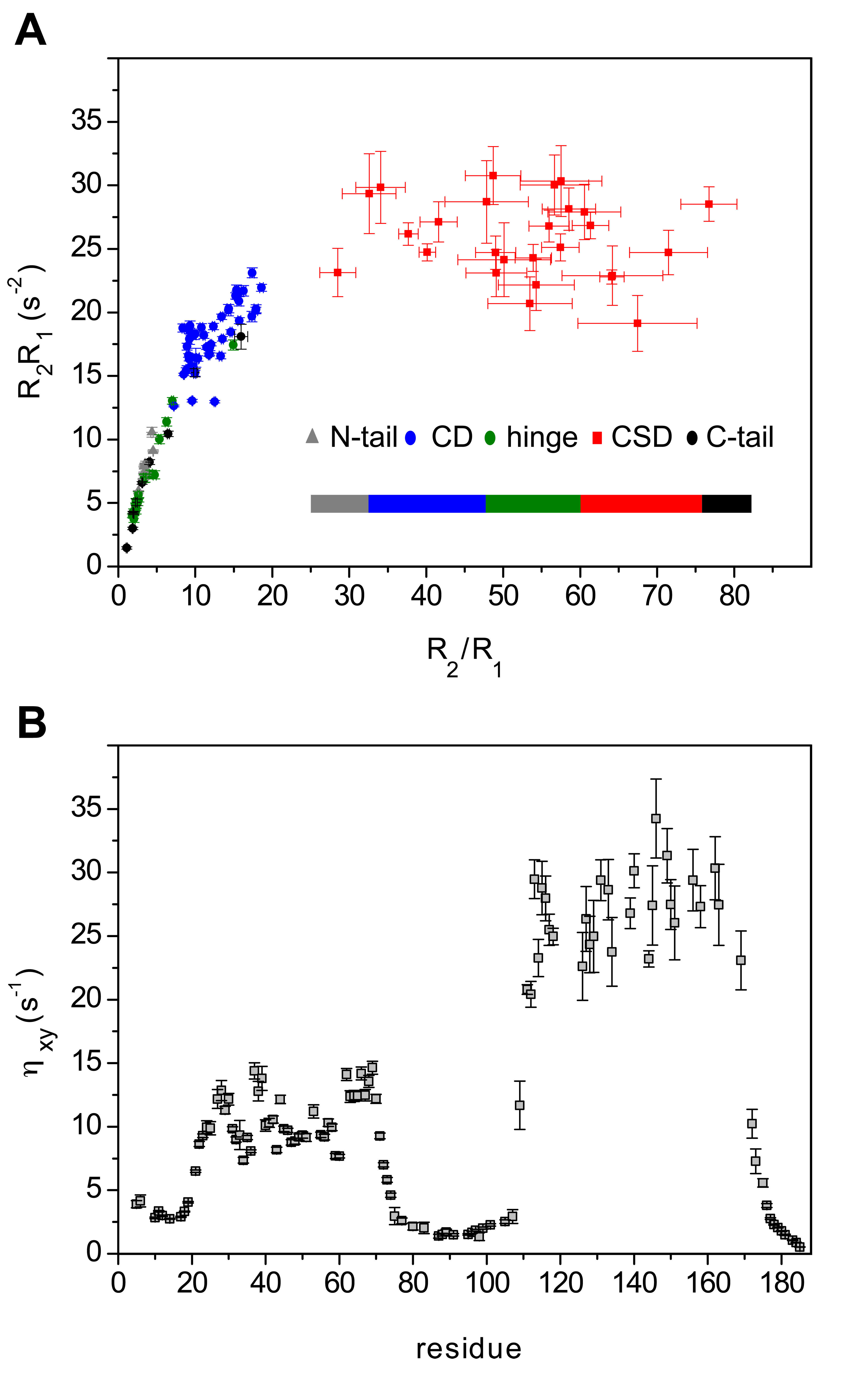

Supplement: Figure S2 — Analysis of 15N spin-relaxation data. A. The anisotropy of global motion can be detected in the plot of R2R1 vs R2/R1 if τc >>1/ωN. The condition is met for CSD where the large distribution of R2/R1 values along constant R2R1 denotes an anisotropic rotational diffusion. B. Transverse cross-correlated relaxation rates (ηxy) of hHP1β as a function of residue number. (TIF) [file pone.0060887.s002.tif]

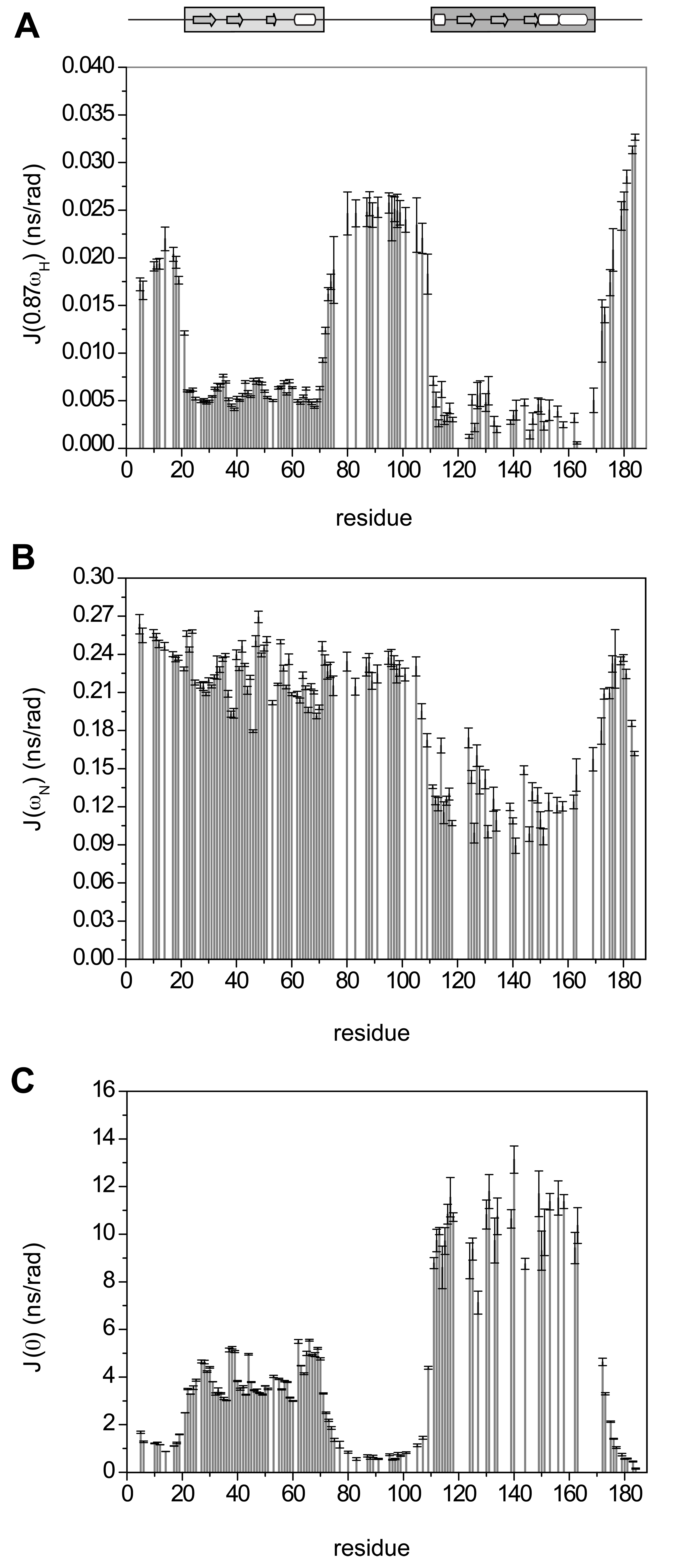

Supplement: Figure S3 — Reduced spectral density mapping. A, B, C. hHP1β 15N spin-relaxation rates were analysed by reduced spectral density mapping. Spectral densities at the effective proton frequency J(0.87ωH) (A), at the 15N frequency J(ωN) (B) and at zero frequency J(0) (C) are shown as a function of residue number. (TIF) [file pone.0060887.s003.tif]

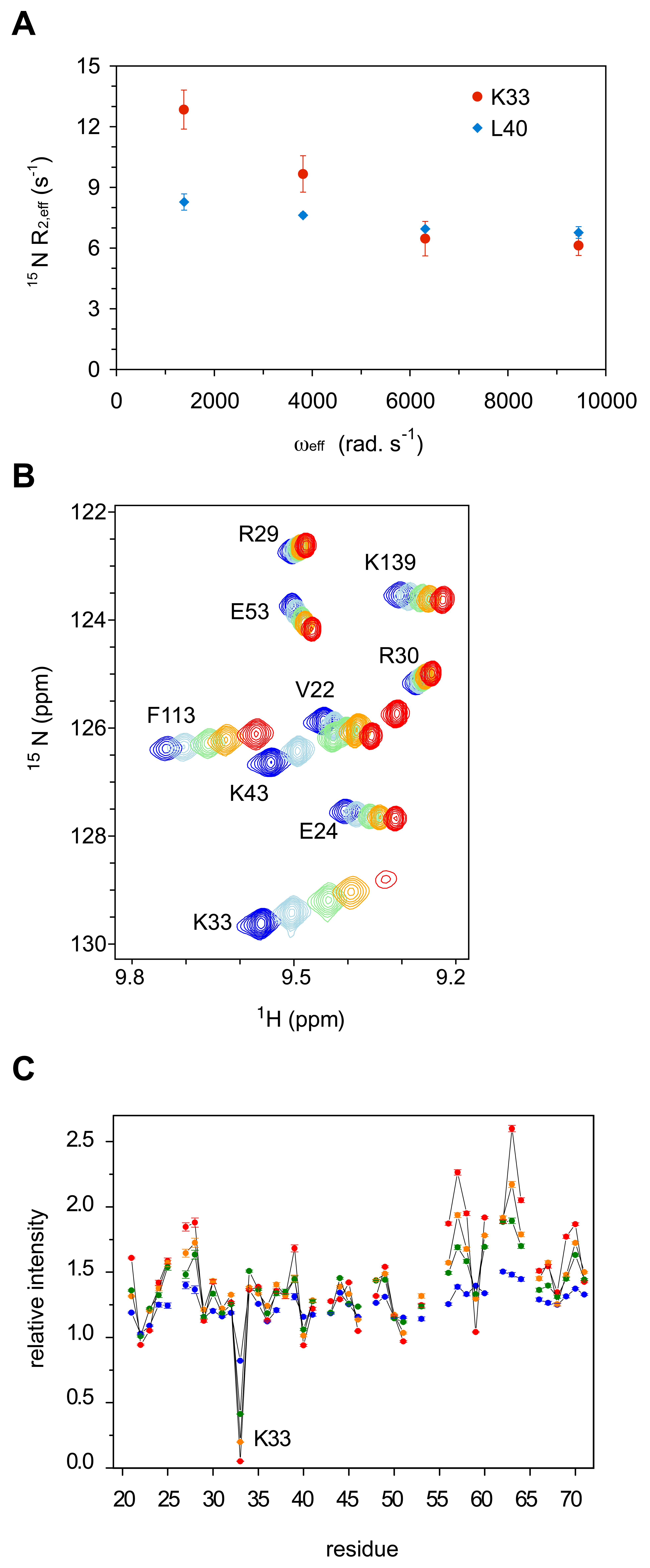

Supplement: Figure S4 — The slow motion of K33. A. Relaxation dispersion profile of K33 in 15N-labelled CD (19–79). Residue L40 serves as control. B. Selected regions of the 1H-15N TROSY-HSQC spectrum of hHP1β at 283 K (blue), 290 K (light blue), 298 K (green), 303 K (yellow), and 310 K (red). Spectra at different temperatures are displayed with equal counter level. K33 shows strong signal broadening at increasing temperature. C. Change of 1H-15N signal intensity with increasing temperature. The signal intensities at 290 K (blue), 298 K (green), 303 K (yellow), and 310 K (red), relative to the intensity at 283 K, are shown versus the CD sequence. (TIF) [file pone.0060887.s004.tif]
